# Evidence-Based Cerebral Vasospasm Surveillance

**DOI:** 10.1155/2013/256713

**Published:** 2013-06-03

**Authors:** Heather Kistka, Michael C. Dewan, J. Mocco

**Affiliations:** Vanderbilt University Medical Center, Department of Neurological Surgery, T-4224 Medical Center North, Nashville, TN 37212, USA

## Abstract

Subarachnoid hemorrhage related to aneurysmal rupture (aSAH) carries significant morbidity and mortality, and its treatment is focused on preventing secondary injury. The most common—and devastating—complication is delayed cerebral ischemia resulting from vasospasm. In this paper, the authors review the various surveillance technologies available to detect cerebral vasospasm in the days following aSAH. First, evidence related to the most common modalities, including transcranial doppler ultrasonography and computed tomography, are reviewed. Continuous electroencephalography and older instruments such as positron emission tomography, xenon-enhanced CT, and single-photon emission computed tomography are also discussed. Invasive strategies including brain tissue oxygen monitoring, microdialysis, thermal diffusion, and jugular bulb oximetry are examined. Lastly, near-infrared spectroscopy, a recent addition to the field, is briefly reviewed. Each surveillance tool carries its own set of advantages and limitations, and the concomitant use of multiple modalities serves to improve diagnostic sensitivity and specificity.

## 1. Introduction

Subarachnoid hemorrhage (SAH) resulting from a ruptured intracranial aneurysm affects approximately 30,000 individuals in USA each year [1]. While many of these patients die before reaching a hospital, the majority are admitted to an intensive care unit where their clinical status can be closely monitored. The damage created by the initial insult of an SAH is irreversible. Therefore, treatment of SAH patients is focused on preventing secondary injury in an effort to minimize morbidity and mortality. The most devastating injury is also among the most common: delayed cerebral ischemia (DCI). Delayed cerebral ischemia affects approximately 30% of all SAH patients and is correlated with a poor outcome as measured by long-term disability and mortality [2, 3]. There have been inconsistencies in the literature regarding the definition of DCI and its frequent precursor, vasospasm. As not all DCI is preceded by vasospasm, this has led to recent efforts to standardize terminology. In general, vasospasm is the result of vasoconstriction or vascular endothelial swelling resulting in a narrowing of the intracranial arteries [4]. On the other hand, DCI is characterized by a new focal neurological deficit, a decrease in the level of consciousness, or radiographic evidence of new infarct [5]. Vasospasm is a radiographic diagnosis that does not necessarily correlate with functional outcome whereas DCI is directly related to morbidity and mortality but may occur in the presence or absence of vasospasm [6]. The diagnosis of vasospasm carries significant importance, as therapy to counteract its deleterious effects can be instituted to protect patients from DCI.

An ideal surveillance method would provide early recognition of vasospasm and allow opportunity to initiate proper therapy prior to the development of symptoms. It would also demonstrate a high positive predictive value so as not to unnecessarily expose patients to the risks of triple-H therapy (hemodilution, hypertension, and hypervolemia), which carries its own inherent risks. This is especially important in patients with particularly severe SAH in whom clinical deterioration is more difficult to detect on the basis of a neurologic exam. This paper will explore the current armamentarium designed for detection of vasospasm recognizing that early intervention decreases the risk of DCI and improves overall survival [7, 8]. Below, the authors review multiple monitoring techniques including transcranial Doppler ultrasonography (TCD), various CT modalities, digital subtraction angiography (DSA), brain tissue oxygenation monitoring (BTO), microdialysis, thermal diffusion monitoring, jugular bulb oximetry, and near infrared spectroscopy (NIRS) and compare their relative sensitivity and specificity as they apply to the clinical setting.

## 2. Surveillance Instruments

### 2.1. Transcranial Doppler Ultrasonography

A diagnostic instrument that provides immediate bedside clinical information in a noninvasive fashion is of obvious utility in SAH patients whose clinical condition is already tenuous. The transcranial Doppler ultrasound (TCD) is a portable and efficient tool for determining cerebral blood flow and has become the most widely used technique for evaluating cerebral vasospasm in the neurocritical care unit [9]. In a meta-analysis by Lysakowski et al. [10], TCD reliably predicted proximal middle cerebral artery (MCA) spasm in 97% of SAH patients. Though specificity was an encouraging 99%, the sensitivity of TCD was found to be 67%. TCD is limited in its ability to obtain consistent measurements in centrally located skull base arteries and several studies have revealed relatively poor sensitivity and specificity in evaluating the anterior cerebral artery and posterior cerebral territories [11, 12]. Despite these shortcomings, clinicians have combined several different TCD-derived calculations to improve diagnostic sensitivity. Gonzalez et al. [13] combined the Lindegaard ratio (middle cerebral artery flow velocity/internal carotid artery flow velocity) and spasm index (TCD velocity/hemispheric cerebral blood flow (CBF)) in a retrospective mathematical model, which increased the sensitivity to 85%, compared to 69–76% for measurements analyzed in isolation. A separate study found that the sensitivity could be increased to 77% by using the ratio of ipsilateral to contralateral MCA mean blood flow volume (mBFV) [14]. Despite its shortfalls, TCD remains the most easily accessible and widely used screening modality available today. When used appropriately, TCD remains a useful screening tool for vasospasm, though providers must exercise caution when using its results to rule out patients with vasospasm.

### 2.2. Computed Tomography

DSA remains the gold standard for diagnosis of cerebral vasospasm and has the added benefit of therapeutic capability. However, DSA is among the most invasive modalities available for vasospasm diagnosis, and leaves much to be desired in the hunt for a low-risk, efficient, and rapid surveillance tool. CT angiography (CTA) has been validated as a first-line tool to recognize and diagnose vasospasm for several years; however, more recently the development of CT perfusion (CTP) scans has been proven to have more diagnostic utility [15]. The appearance of vasospasm on CTA is highly reproducible; however, the quality of images depends on the technique of dye administration and the limitation of motion artifact. Additionally, CTA tends to overestimate the size of larger blood vessels, thereby creating a more severe appearance of vasospasm than is actually present [12]. This can potentially lead to detrimental effects if patients are inappropriately subjected to triple-H therapy. Additionally, it is important to recall that angiographic vasospasm is not directly correlated with DCI and may occur in isolation without any adverse clinical effects. Therefore it remains important to identify patients in whom DCI and vasospasm are simultaneously present, indicating they could benefit from triple-H therapy.

Recent advances in CTP imaging have produced promising results. Retrospective analysis of radiographs obtained 6–8 days after hemorrhage reveals similar sensitivity (80% and 73%), specificity (67% and 75%), and positive predictive value (90% and 92%) for CTP and DSA, respectively [16]. These results have been validated in a prospective trial in which analysis of CBF and mean transit time (MTT) on CTP were 93% and 88% accurate, respectively, in diagnosing DCI [17]. Additionally, a meta-analysis found similar sensitivity and specificity when comparing CTA and CTP [18]. However, it has also been noted that the accuracy of both CTA and DSA decreases when evaluating smaller caliber vessels. CTP, therefore, may play a critical complimentary role in evaluating distal vasculature. Additionally, a decrease in CBF and prolongation of MTT on baseline CTP performed as a baseline within 3 days following SAH have been found to be >90% specific for the development of vasospasm [19]. Similarly, early results suggest that CTP may be a reliable predictor of DCI. In a recent study involving 96 patients with SAH, CTP predicted DCI with 81% sensitivity and 83% specificity [20]. Further prospective studies are needed, but this is a promising, relatively new technique to identify and potentially treat the subset of patients at highest risk for vasospasm early in their hospital course.

### 2.3. Other Imaging Techniques to Evaluate Cerebral Perfusion

While CTP is perhaps the most commonly employed modality, several other methods are quickly gaining rapport among cerebrovascular surgeons for prediction of vasospasm. Perfusion-weighted magnetic resonance imaging (MRI) has been demonstrated to reveal small regions of early ischemic injury, indicating territories suffering vasospasm [21]. Decreases in relative cerebral blood volume (CBV) and mean transit time on diffusion-weighted MRI assist in the selection of patients who benefit from triple-H therapy [22].

Several older modalities, including positron emission tomography (PET), xenon-enhanced CT (Xe-CT), and single-photon emission computed tomography (SPECT) are available for evaluation of brain tissue distal to vessels undergoing vasospasm [23]. PET provides physiologic data by demonstrating the brain's effort to maintain an adequate cerebral metabolic rate in the setting of SAH [24]. Evidence established by PET shows increase oxygen extraction and increased blood volume, seemingly related to microarterial dilatation in the days following aneurysm rupture [25]. SPECT utilizes a glial and neuronal-metabolized radioisotope to detect areas of relatively high tissue oxygen demand [26]. Importantly, the SPECT technique is not a quantitative measure, but rather one that relies on the relative differences in cerebral blood flow between two or more regions [27]. Thus, diffuse or bihemispheric hypoperfusion could be misinterpreted as a normal perfusion study, as analyzed by SPECT [28]. Furthermore, while regions of hypoperfusion correlate well with ischemic deficits, the resource-burden and low resolution relative to other technologies limit its widespread and routine use [29]. Another surveillance modality relying on CT technology, Xenon-enhanced CT, may not only identify ischemic tissue, but also has been shown to confirm areas salvaged by medical or endovascular intervention [30]. Upon its introduction, the primary advantage of Xe-CT relative to its contemporary counterparts was the ability to detect very early flow derangements (within two hours or less) [31]. Today, the widespread availability of and familiarity with CTP has rendered Xe-CT largely obsolete.

### 2.4. Continuous Electroencephalogram

Continuous EEG (cEEG) monitoring is an attractive surveillance method owing to its sensitivity in detecting cerebral ischemia and the fact that it is currently the only widely available continuous monitor other than the physical exam. Additionally, patients with SAH have a 19% risk of developing seizures [32]. Continuous EEG has the added benefit of diagnosing subclinical seizures in patients whose neurologic exam cannot reliably distinguish seizure activity from ischemia-related global cerebral insult.

Many EEG parameters have been employed to detect ischemia. One prospective study of relative alpha wave activity demonstrated that a decrease in such activity detected cerebral vasospasm with 100% sensitivity in an average of 2.9 days earlier than TCD or angiography [33]. It was not, however, able to differentiate decreased activity due to vasospasm from that due to increased intracranial pressure yielding a specificity of only 50%. Therefore, EEG may represent a reliable screening tool, but its initial findings must be validated by additional measures. In the absence of confirmation of vasospasm via TCD or CTA, the low positive predictive value leads to a therapeutic dilemma in the case where one must decide if the elevation is an early precursor to vasospasm that should be proactively treated or a false positive. Utilizing quantitative analysis with cEEG allows differentiation of vasospasm from seizure activity with variable reliability, but generally earlier than imaging modalities such as CT or MRI [32]. However, The labor intensive process of applying electrodes which must be removed and reapplied for CT or MRI and the difficulty of analyzing the data in real time without an electroencephalographer at all times have limited the widespread use of this technique. Nevertheless, cEEG is an ideal instrument for those patients with high-grade SAH and poor neurologic exam in whom there is clinical suspicion for both seizure activity and ischemia.

### 2.5. Invasive Tissue Monitoring: Brain Tissue Oxygen Monitoring and Microdialysis

Both brain tissue oxygen (PtiO_2_) monitoring and microdialysis require implantation of a probe in a region of interest and provide data regarding changes in the surrounding microenvironment at frequent time intervals. In 2000, the first PtiO_2_ monitoring in SAH demonstrated lower cerebral tissue pH and higher PCO_2_ compared to controls [34], and a subsequent investigation indicated a strong correlation between PtiO_2_ and vasospasm [35]. A separate study suggests that PtiO_2_ pressure reactivity is a reliable indicator of impaired autoregulation and, by proxy, vasospasm effect [36]. These studies, and similar investigations involving PtiO_2_, are plagued by a small sample size—not to mention the innate invasiveness of PtiO_2_—limiting their universal adoption. Moreover, exploration by some of the relationship between pressure- and oxygen-related indices of vasospasm and clinical outcome has revealed a discordant relationship, calling into question the utility and reliability of PtiO_2_ [37].

Cerebral microdialysis is capable of measuring local levels of a variety of metabolic markers including glutamate [38–40], lactate [38, 41], and glucose [41] and relating values to areas of ischemia and clinical outcome. In one study, microdialysis has been shown to be 89% specific for ischemia, detecting dramatic changes in concentrations of lactate, glucose, and glutamate levels before a patient becomes symptomatic [42]. Higher levels of lactate, nitrite, and taurine, all measured via microdialysis, are predictive of poor neurologic outcome after SAH [43]. Despite these results, the practical limitations of this technique have curtailed its more widespread use. It is an invasive monitor that must be placed in a region of brain with viable tissue near the site of rupture. The growing popularity of endovascular treatments obviating the dependence upon open craniotomies necessitates that the majority of monitors be placed at the bedside where proper placement is considerably more difficult and dangerous. Additionally, the probe is only able to monitor a small region of interest thus decreasing the sensitivity and limiting its utility in monitoring a condition that can have patchy or global effects.

### 2.6. Invasive Cerebral Blood Flow Monitoring: Thermal Diffusion and Jugular Bulb Oximetry

Continuous monitoring of cerebral blood flow is another means to detect vasospasm and its effects on tissue [44, 45]. While TCD can monitor this at a discrete moment in time, reliable continuous monitors may play a crucial role in vasospasm detection and management as well as predicting which patients will suffer from DCI. This can be achieved locally via thermal diffusion probes and globally via jugular bulb oximetry.

Much like in microdialysis, a thermal diffusion probe may be inserted into the brain via a burr hole into the white matter of a region of interest thought to be at risk for ischemia. The probe contains 2 gold sensors; one receives the heat emitted by the other [44]. The thermal difference between the plates varies with the CBF. This data can be gathered continuously in real time. Although the literature on this technique is sparse, Vajkoczy et al. found that CBF continues to decrease after posthemorrhage day four in patients with vasospasm even as the CPP rises during that time [45]. In their small series, a regional CBF of 15 mg/100 g/min had 90% sensitivity and 75% specificity for vasospasm when using a single probe. This was more reliable than TCD and detected symptomatic vasospasm on average three days earlier than conventional methods. Despite these promising preliminary results, the difficulty of implanting the probe has limited its widespread use. Additionally, like in microdialysis, a thermal diffusion probe is able to monitor only a single region of brain and therefore its efficacy is predicated upon appropriate selection of a region of interest.

Jugular bulb oximetry offers a global view of cerebral perfusion but is less precise. This technique involves the insertion of an oxygen saturation probe high into the jugular vein above the facial vein, allowing sampling from intracranial circulation [46]. The resulting venous oxygen saturation can then be subtracted from the arterial oxygen saturation to yield the cerebral oxygen extraction (CEO_2_ or AVDO_2_). Results from a small sample [47] suggest that this value increases significantly more than 24 hours prior to the onset of symptomatic vasospasm. These levels normalized after institution of triple-H therapy and corresponded to symptomatic improvement in patients. Additionally, subjects who experienced clinical vasospasm had significantly higher AVDO_2_ at baseline than those who did not [47]. These preliminary results seem promising but have not been validated in larger studies. The technique is relatively simple but does expose patients to the risk of thrombus from the catheter, and the literature remains inconclusive on whether or not laterality catheter placement affects the integrity of the results.

### 2.7. Near-Infrared Spectroscopy

The most recent advancement in the evaluation of cerebral blood flow dynamics, NIRS, utilizes near-infrared light to penetrate the skull and provide a quantitative measurement of hemoglobin concentration and cortical oxygen saturation (CoSO2) in the underlying cortex. NIRS has been shown to reliably assess cerebral autoregulation in SAH patients in a continuous, real-time fashion [48–50]. In a small study by Yokose et al. [51], a 3.9–6.4% decrease in CoSO2 in the MCA region detected by daily measurements was found to be 100% sensitive and 85.7% specific for vasospasm confirmed with CTA. This was considerably more accurate than the results obtained via TCD leading to the conclusion that this is a promising new technique that could replace TCD as the standard noninvasive monitoring of SAH patients. Moreover, unlike TCD, NIRS is continuous, requires little clinical attention, and is optimal for patients who may be agitated or otherwise uncooperative. Despite these favorable attributes and encouraging initial results, larger, prospective studies are necessary to validate this technique before it can safely be adopted into widespread use.

## 3. Conclusion

Overall, vasospasm and DCI remain a challenge to detect in patients with a poor neurologic exam. The logistical difficulties encountered in critically ill patients are compounded by a body of the literature which has been plagued by inconsistent definitions of clinical entities which make interpreting and comparing data difficult. Older modalities such as SPECT and Xe-CT may be impractical in today's clinical setting; however, their contribution to our understanding of cerebral blood flow and metabolism cannot be overlooked. Similarly, while PtiO_2_ and microdialysis are rarely employed clinically, they continue to play an important role in the laboratory and promise to provide answers to remaining questions about the cerebral microenvironment following SAH. Although imperfect, TCD maintains a critical role in the detection of vasospasm. Continuous EEG has demonstrated sensitivity in detecting ischemia, though must be partnered with other surveillance modalities to achieve reliable clinical utility. Both EEG and TCD may be combined with clinical exam and increasingly with CT perfusion imaging to identify patients who are likely to have adverse effects related to vasospasm. Future prospective analysis should focus on the utility of early CTP and the development of NIRS to provide early, noninvasive detection of ischemic injury.

## References

[1] King JT (1997). Epidemiology of aneurysmal subarachnoid hemorrhage. *Neuroimaging Clinics of North America*.

[2] Kassell NF, Sasaki T, Colohan ART, Nazar G (1985). Cerebral vasospasm following aneurysmal subarachnoid hemorrhage. *Stroke*.

[3] Vergouwen MDI, Participants in the International Multi-Disciplinary Consensus Conference on the Critical Care Management of Subarachnoid Hemorrhage (2011). Vasospasm versus delayed cerebral ischemia as an outcome event in clinical trials and observational studies. *Neurocritical Care*.

[4] Hughes JT, Schianchi PM (1978). Cerebral artery spasm. A histological study at necropsy of the blood vessels in cases of subarachnoid hemorrhage. *Journal of Neurosurgery*.

[5] Frontera JA, Fernandez A, Schmidt JM (2009). Defining vasospasm after subarachnoid hemorrhage: what is the most clinically relevant definition?. *Stroke*.

[6] Vergouwen MDI, Vermeulen M, van Gijn J (2010). Definition of delayed cerebral ischemia after aneurysmal subarachnoid hemorrhage as an outcome event in clinical trials and observational studies: proposal of a multidisciplinary research group. *Stroke*.

[7] Muizelaar JP, Becker DP (1986). Induced hypertension for the treatment of cerebral ischemia after subarachnoid hemorrhage. Direct effect on cerebral blood flow. *Surgical Neurology*.

[8] Darby JM, Yonas H, Marks EC, Durham S, Snyder RW, Nemoto EM (1994). Acute cerebral blood flow response to dopamine-induced hypertension after subarachnoid hemorrhage. *Journal of Neurosurgery*.

[9] Deb S, Gogos AJ, Drummond KJ, Teddy PJ (2012). The role of transcranial Doppler ultrasound monitoring in patients with aneurysmal subarachnoid haemorrhage. *Journal of Clinical Neuroscience*.

[10] Lysakowski C, Walder B, Costanza MC, Tramèr MR (2001). Transcranial Doppler versus angiography in patients with vasospasm due to a ruptured cerebral aneurysm: a systematic review. *Stroke*.

[11] Carrera E, Schmidt JM, Oddo M (2009). Transcranial Doppler for predicting delayed cerebral ischemia after subarachnoid hemorrhage. *Neurosurgery*.

[12] Washington CW, Zipfel GJ, Participants in the International Multi-Disciplinary Consensus Conference on the Critical Care Management of Subarachnoid Hemorrhage (2011). Detection and monitoring of vasospasm and delayed cerebral ischemia: a review and assessment of the literature. *Neurocritical Care*.

[13] Gonzalez NR, Boscardin WJ, Glenn T, Vinuela F, Martin NA (2007). Vasospasm probability index: a combination of transcranial Doppler velocities, cerebral blood flow, and clinical risk factors to predict cerebral vasospasm after aneurysmal subarachnoid hemorrhage. *Journal of Neurosurgery*.

[14] Nakae R, Yokota H, Yoshida D, Teramoto A (2011). Transcranial Doppler ultrasonography for diagnosis of cerebral vasospasm after aneurysmal subarachnoid hemorrhage: mean blood flow velocity ratio of the ipsilateral and contralateral middle cerebral arteries. *Neurosurgery*.

[15] Dankbaar JW, de Rooij NK, Velthuis BK, Frijns CJM, Rinkel GJE, van der Schaaf IC (2009). Diagnosing delayed cerebral ischemia with different CT modalities in patients with subarachnoid hemorrhage with clinical deterioration. *Stroke*.

[16] Killeen RP, Mushlin AI, Johnson CE (2011). Comparison of CT perfusion and digital subtraction angiography in the evaluation of delayed cerebral ischemia. *Academic Radiology*.

[17] Sanelli PC, Ugorec I, Johnson CE (2011). Using quantitative CT perfusion for evaluation of delayed cerebral ischemia following aneurysmal subarachnoid hemorrhage. *American Journal of Neuroradiology*.

[18] Greenberg ED, Gold R, Reichman M (2010). Diagnostic accuracy of CT angiography and CT perfusion for cerebral vasospasm: a meta-analysis. *American Journal of Neuroradiology*.

[19] Sanelli PC, Jou A, Gold R (2011). Using CT perfusion during the early baseline period in aneurysmal subarachnoid hemorrhage to assess for development of vasospasm. *Neuroradiology*.

[20] Sanelli PC, Anumula N, Johnson CE (2013). Evaluating CT perfusion using outcome measures of delayed cerebral ischemia in aneurysmal subarachnoid hemorrhage. *American Journal of Neuroradiology*.

[21] Rordorf G, Koroshetz WJ, Copen WA (1999). Diffusion- and perfusion-weighted imaging in vasospasm after subarachnoid hemorrhage. *Stroke*.

[22] Sorensen AG, Copen WA, Østergaard L (1999). Hyperacute stroke: simultaneous measurement of relative cerebral blood volume, relative cerebral blood flow, and mean tissue transit time. *Radiology*.

[23] Lad SP, Guzman R, Kelly ME (2006). Cerebral perfusion imaging in vasospasm. *Neurosurgical Focus*.

[24] Powers WJ, Grubb RL, Baker RP (1985). Regional cerebral blood flow and metabolism in reversible ischemia due to vasospasm; determination by positron emission tomography. *Journal of Neurosurgery*.

[25] Kawamura S, Sayama I, Yasui N, Uemura K (1992). Sequential changes in cerebral blood flow and metabolism in patients with subarachnoid haemorrhage. *Acta Neurochirurgica*.

[26] Walovitch RC, Cheesman EH, Maheu LJ, Hall KM (1994). Studies of the retention mechanism of the brain perfusion imaging agent 99mTc-bicisate (99mTc-ECD). *Journal of Cerebral Blood Flow and Metabolism*.

[27] Davis SM, Andrews JT, Lichtenstein M, Rossiter SC, Kaye AH, Hopper J (1992). Correlations between cerebral arterial velocities, blood flow, and delayed ischemia after subarachnoid hemorrhage. *Stroke*.

[28] Davis S, Andrews J, Lichtenstein M (1990). A single-photon emission computed tomography study of hypoperfusion after subarachnoid hemorrhage. *Stroke*.

[29] Firlik KS, Kaufmann AM, Firlik AD, Jungreis CA, Yonas H (1999). Intra-arterial papaverine for the treatment of cerebral vasospasm following aneurysmal subarachnoid hemorrhage. *Surgical Neurology*.

[30] Yonas H, Sekhar L, Johnson DW, Gur D (1989). Determination of irreversible ischemia by xenon-enhanced computed tomographic monitoring of cerebral blood flow in patients with symptomatic vasospasm. *Neurosurgery*.

[31] Bouma GJ, Muizelaar JP (1993). Evaluation of regional cerebral blood flow in acute head injury by stable xenon-enhanced computerized tomography. *Acta Neurochirurgica*.

[32] Claassen J, Mayer SA, Hirsch LJ (2005). Continuous EEG monitoring in patients with subarachnoid hemorrhage. *Journal of Clinical Neurophysiology*.

[33] Vespa PM, Nuwer MR, Juhász C (1997). Early detection of vasospasm after acute subarachnoid hemorrhage using continuous EEG ICU monitoring. *Electroencephalography and Clinical Neurophysiology*.

[34] Charbel FT, Du X, Hoffman WE, Ausman JI (2000). Brain tissue PO_2_, PCO_2_, and pH during cerebral vasospasm. *Surgical Neurology*.

[35] Khaldi A, Zauner A, Reinert M, Woodward JJ, Bullock MR (2001). Measurement of nitric oxide and brain tissue oxygen tension in patients after severe subarachnoid hemorrhage. *Neurosurgery*.

[36] Jaeger M, Schuhmann MU, Soehle M, Nagel C, Meixensberger J (2007). Continuous monitoring of cerebrovascular autoregulation after subarachnoid hemorrhage by brain tissue oxygen pressure reactivity and its relation to delayed cerebral infarction. *Stroke*.

[37] Barth M, Woitzik J, Weiss C (2010). Correlation of clinical outcome with pressure-, oxygen-, and flow-related indices of cerebrovascular reactivity in patients following aneurysmal SAH. *Neurocritical Care*.

[38] Enblad P, Valtysson J, Andersson J (1996). Simultaneous intracerebral microdialysis and positron emission tomography in the detection of ischemia in patients with subarachnoid hemorrhage. *Journal of Cerebral Blood Flow and Metabolism*.

[39] Persson L, Valtysson J, Enblad P (1996). Neurochemical monitoring using intracerebral microdialysis in patients with subarachnoid hemorrhage. *Journal of Neurosurgery*.

[40] Schulz MK, Wang LP, Tange M, Bjerre P (2000). Cerebral microdialysis monitoring: determination of normal and ischemic cerebral metabolisms in patients with aneurysmal subarachnoid hemorrhage. *Journal of Neurosurgery*.

[41] Nilsson OG, Brandt L, Ungerstedt U, Säveland H (1999). Bedside detection of brain ischemia using intracerebral microdialysis: subarachnoid hemorrhage and delayed ischemic deterioration. *Neurosurgery*.

[42] Unterberg AW, Sakowitz OW, Sarrafzadeh AS, Benndorf G, Lanksch WR (2001). Role of bedside microdialysis in the diagnosis of cerebral vasospasm following aneurysmal subarachnoid hemorrhage. *Journal of Neurosurgery*.

[43] Staub F, Graf R, Gabel P, Köchling M, Klug N, Heiss W (2000). Multiple interstitial substances measured by microdialysis in patients with subarachnoid hemorrhage. *Neurosurgery*.

[44] Steiner LA, Andrews PJD (2006). Monitoring the injured brain: ICP and CBF. *British Journal of Anaesthesia*.

[45] Vajkoczy P, Horn P, Thome C, Munch E, Schmiedek P (2003). Regional cerebral blood flow monitoring in the diagnosis of delayed ischemia following aneurysmal subarachnoid hemorrhage. *Journal of Neurosurgery*.

[46] von Helden A, Schneider GH, Unterberg A, Lanksch WR (1993). Monitoring of jugular venous oxygen saturation in comatose patients with subarachnoid haemorrhage and intracerebral haematomas. *Acta Neurochirurgica*.

[47] Heran NS, Hentschel SJ, Toyota BD (2004). Jugular bulb oximetry for prediction of vasospasm following subarachnoid hemorrhage. *Canadian Journal of Neurological Sciences*.

[48] Mutoh T, Ishikawa T, Suzuki A, Yasui N (2010). Continuous cardiac output and near-infrared spectroscopy monitoring to assist in management of symptomatic cerebral vasospasm after subarachnoid hemorrhage. *Neurocritical Care*.

[49] Poon WS, Wong GKC, Ng SCP (2010). The quantitative time-resolved near infrared spectroscopy (TR-NIRs) for bedside cerebrohemodynamic monitoring after aneurysmal subarachnoid hemorrhage: can we predict delayed neurological deficits?. *World Neurosurgery*.

[50] Zweifel C, Castellani G, Czosnyka M (2010). Continuous assessment of cerebral autoregulation with near-infrared spectroscopy in adults after subarachnoid hemorrhage. *Stroke*.

[51] Yokose N, Sakatani K, Murata Y (2010). Bedside monitoring of cerebral blood oxygenation and hemodynamics after aneurysmal subarachnoid hemorrhage by quantitative time-resolved near-infrared spectroscopy. *World Neurosurgery*.

